# Feasibility study on the introduction of Micro-CT technology for the identification of *Radix Bupleuri* and its adulterants

**DOI:** 10.3389/fphar.2024.1347316

**Published:** 2024-02-28

**Authors:** Kehong Chen, Gong Chen, Zhelong Zhuang, Shouhua Luo, Jiaming Liu, Guorong Liu

**Affiliations:** ^1^ School of Artificial Intelligence and Information Technology, Nanjing University of Chinese Medicine, Nanjing, Jiangsu, China; ^2^ Affiliated Hospital of Nanjing University of Traditional Chinese Medicine, Nanjing, Jiangsu, China; ^3^ School of Biological & Medical Engineering, Southeast University, Nanjing, Jiangsu, China

**Keywords:** Micro-CT technology, *Radix Bupleuri*, microstructure, identification, 3D reconstruction, data augmentation, ResNeXt

## Abstract

**Background:** *Radix Bupleuri*, a kind of Chinese herbal medicine with great clinical use, is often confused with its adulterants, and it is difficult to identify it without certain knowledge. The existing identification methods have their own drawbacks, so a new method is needed to realize the identification of *Radix Bupleuri* and its adulterants.

**Methods:** We used Micro Computed Tomography (Micro-CT) to perform tomography scans on *Radix Bupleuri* and its adulterants, performed data screening and data correction on the obtained DICOM images, and then applied 3D reconstruction, data augmentation, and ResNext deep learning model for the classification study.

**Results:** The DICOM images after data screening, data correction, and 3D reconstruction can observe the differences in the microstructure of *Radix Bupleuri* and its adulterants, thus enabling effective classification and analysis. Meanwhile, the accuracy of classification using the ResNext model reached 75%.

**Conclusion:** The results of this study showed that Micro-CT technology is feasible for the authentication of *Radix Bupleuri*. The pre-processed and 3D reconstructed tomographic images clearly show the microstructure and the difference between *Radix Bupleuri* and its adulterants without damaging the internal structure of the samples. This study concludes that Micro-CT technology provides important technical support for the reliable identification of *Radix Bupleuri* and its adulterants, which is expected to play an important role in the quality control and clinical application of herbs.

## 1 Introduction

According to the *Chinese Pharmacopoeia*, *Radix Bupleuri,* also called “Chaihu” in Chinese, is derived from the dried roots of *Bupleurum chinense DC*. and *Bupleurum scorzonerifolium Willd*. (Editorial Committee of Chinese Pharmacopoeia, 2020). According to different characters, it is called “North-chaihu” and “South-chaihu,” which has the effect of evacuating and reducing fever, soothing the liver and relieving depression, and lifting Yang qi. It is often used to treat cold and fever, cold and heat exchange, chest and hypogastric pain, menstrual irregularity, uterine prolapse, and anal prolapse (Yang et al., 2017). In recent years, with a deeper insight into the relevant research, it has been found that *Radix Bupleuri* has anti-inflammatory, antiviral, liver and gallbladder protective effects, prevents and treats cancer, balances different organs and energy in the body, strengthens the digestive tract, improves the function of the liver and circulatory system, and relieves liver tension, among others (Ma et al., 2016). However, the safety and effectiveness of the use of *Radix Bupleuri* are challenging to guarantee for two reasons: first, because the efficacy of *Radix Bupleuri* is significant, some merchants illegally mix the products for some profits, which seriously affects the regular operation of the medicinal material market and dramatically undermines the safety and effectiveness of the use of *Radix Bupleuri*; second, the seeds of *Radix Bupleuri* tend to be mixed with those of its adulterants when planting, and it is difficult to distinguish after growth. *Radix Bupleuri* and its adulterants share a similar morphological appearance, but the chemicals they contain differ considerably, including *Bupleurum longiradiatum Turcz.* with toxic ingredients and *Bupleurum hamiltonii Balak* with almost undetectable saikosaponin contents (Chao et al., 2014). More seriously, *B. longiradiatum Turcz.* has been reported by Heilongjiang Institute for Drug Control for mistakenly confusing commercial *Radix Bupleuri* preparations and causing poisoning incidents (Shen et al., 2024). Therefore, identifying and classifying *Radix Bupleuri* and its adulterants are particularly important in order to prevent similar incidents.

At present, the identification methods for *Radix Bupleuri* and its adulterants include character identification (Editorial Committee of Chinese Pharmacopoeia, 2020), DNA barcode identification (Feng et al., 2015), TLC identification (Yen et al., 1991), HPLC identification (Qian, 2012), and microscopic identification (Miao et al., 2017). Existing identification methods have their advantages and disadvantages: character identification is simple but requires rich knowledge of traditional Chinese medicine identification; DNA barcode identification, thin-layer chromatography identification, HPLC identification, and microscopic identification features high accuracy, but the operation is too complicated. Therefore, it is of great importance to introduce Micro Computed Tomography (Micro-CT) technology as a new method for *Radix Bupleuri* identification.

Micro-CT technology differs from clinical CT technology in that the X-ray emission tube of Micro-CT is a micro-focus X-ray tube, that can realize the imaging of small/micro-volume samples at a resolution level of 1–100 μm. It has a good “microscopic effect,” which can be applied to show the nuanced structural differences in small samples. Micro-CT is widely recognized as the preferred method for studying the microstructure, spatial volume, and structural changes of bones. Meanwhile, Micro-CT technology is an advanced, non-destructive 3D imaging technology that has been researched and applied in many fields, such as medicine, geological research, materials, and agriculture (Wong et al., 2010; van Raamsdonk et al., 2019; Feng et al., 2021; Ma et al., 2021; Xie et al., 2021). Different from ordinary clinical CT, it is a technology used for scanning and analyzing images of living small animals, various complex tissues and related soft tissues with a micro-focus X-ray tube. However, Micro-CT technology is rarely used in plants and Chinese medicine (Starosolski et al., 2015; Su and Xiao, 2020). Micro-CT technology can scan *Radix Bupleuri* and its adulterants products, and corresponding DICOM images are obtained and studied. In addition, Micro-CT technology can not only accurately obtain the internal microstructure of traditional Chinese medicine (TCM) decoction pieces but also preserve the integrity of the sample to the greatest extent to facilitate future follow-up studies of the sample (Zhang et al., 2020).

In addition, Micro-CT can be combined with 3D reconstruction technology to obtain clear and precise 3D deconstructed images of the test specimen. The FDK algorithm (Feldkamp-Davis-Kress) used in this study is the most commonly used reconstruction algorithm for CT images. The FDK algorithm, first proposed by Feldkamp, Davis, and Kress in 1984, is an approximate reconstruction algorithm for cone-beam CT circular-trajectory scans, and has been the most practical algorithm for cone-beam circular-trajectory reconstruction to date (Feldkamp et al., 1984). DICOM images after FDK 3D reconstruction are able to generate high-quality 3D reconstructed images, reducing artifacts and image distortions and improving the spatial resolution and geometric accuracy of the images. At the same time, the image after FDK 3D reconstruction can retain multi-layered information about the structure of the dissection, preserving the 2D slice features while permitting the formation of a 3D model to study its 3D structural features if needed.

What’s more, ResNext (Residual Next) used in this study is a deep neural network architecture that extends and improves on ResNet (Residual Network) (Xie et al., 2016). The design goal of ResNext is to increase the expressive power of the model while maintaining its simplicity, thus improving the performance in various computer vision tasks. ResNext, we introduce the concept of “cardinality” to the idea of residual learning in ResNet. In ResNext, the branches within each residual block are divided into multiple paths, each with the same convolutional layers but different weight parameters. This structure allows the network to learn features in multiple dimensions, and the complexity of the model can be increased by increasing the number of cardinalities.

Hence, this study aims to provide a new method for the authenticity identification of *Radix Bupleuri* and its adulterants products by analyzing and identifying DICOM images obtained after Micro-CT imaging. The application of Micro-CT technology provides us with a unique perspective that allows us to explore the microstructure of *Radix Bupleuri* and its adulterants in depth, thus discovering small but important differences between them. This method not only improves the quality control of *Radix Bupleuri* herbs, but also is expected to play a key role in the field of Chinese herbal medicine identification to ensure the safety of medication. By combining DICOM image analysis and advanced 3D reconstruction techniques and deep learning techniques, we will be able to achieve reliable classification and identification of *Radix Bupleuri* and its adulterants, providing new tools and perspectives for quality management and monitoring of Chinese herbal medicines. This study is expected to bring innovative advances in quality control and applications in the field of Chinese herbal medicine.

## 2 Materials and methods

### 2.1 Experimental materials

**FIGURE 1:**
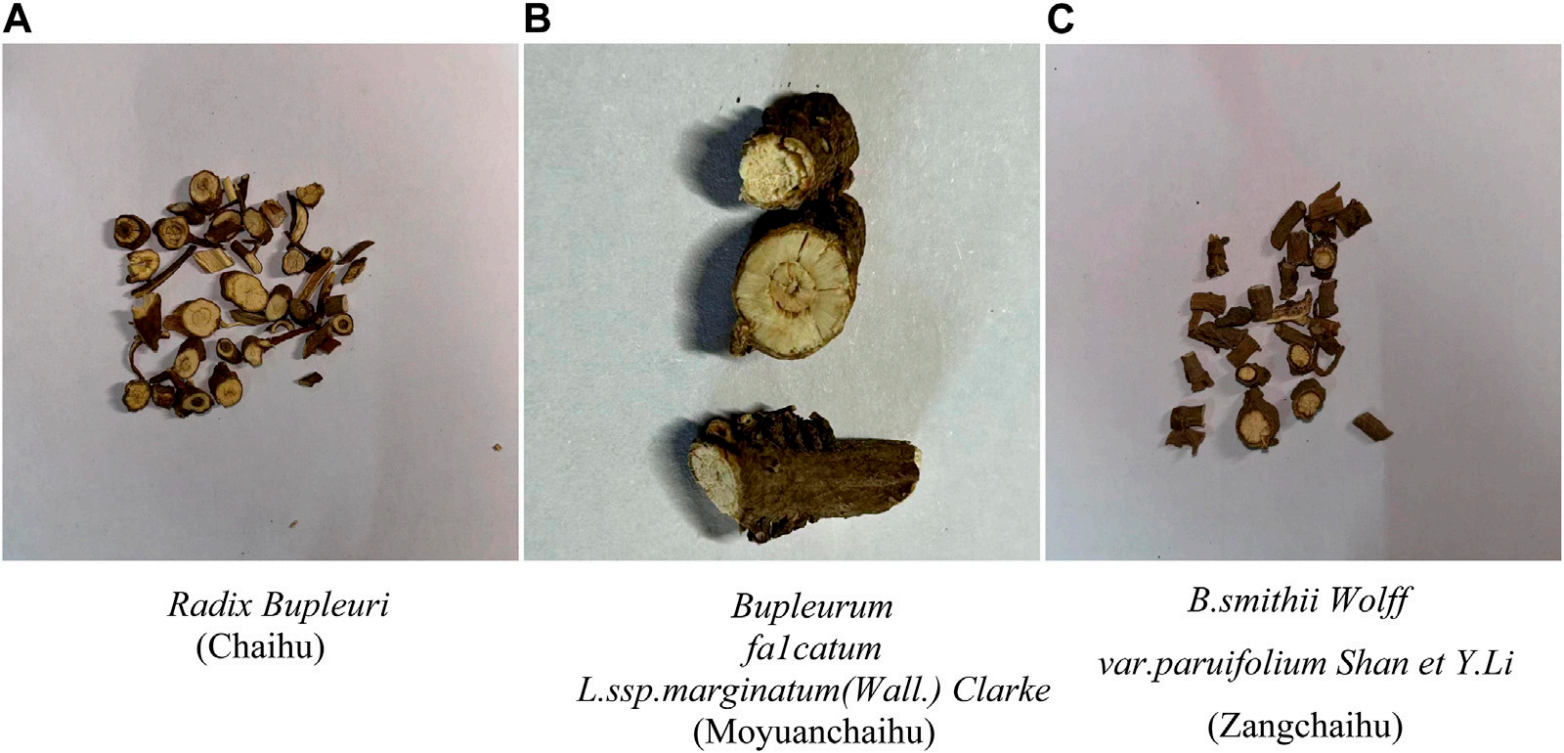
Optical photographs of three types of *Radix Bupleuri*. **(A)** *Shows the optical image of Radix Bupleuri* (Chaihu). **(B)** Shows the microstructure of the *Bupleurum fa1catum L. ssp.marginatum(Wall.) Clarke* (Moyuanchaihu). **(C)** Shows the microstructure of *B. smithii Wolff var. paruifolium Shan et Y.Li* (Zangchaihu). From the above images, it can be observed that the three types of *Radix Bupleuri* and its adulterants are more difficult to be classified and identified under normal visual observation, including the cross-section of the external features.

This study collected nine samples of *Radix Bupleuri* (Chaihu), *Bupleurum fa1catum L. ssp.marginatum (Wall.) Clarke* (Moyuanchaihu), and *B. smithii Wolff var. paruifolium Shan et Y.Li* (Zangchaihu). Among them, Moyuanchaihu and Zangchaihu have been identified. Jiangsu Provincial Hospital of Traditional Chinese Medicine identified all samples. Optical photographs of three types of *Radix Bupleuri* and its adulterants used in this study are shown in Figure 1.

### 2.2 Equipment and data collection

The Micro-CT machine used in this study was provided by Southeast University. In addition, the hardware information of the experimental platform used in this study is as follows: 13th Gen Intel(R) Core (TM) i9-13900KF, NVIDIA GeForce RTX 4090 24 GB. The software information of the experimental platform used in this study is as follows: the operating system is Windows 10, the programming language is Python 3.7.12, and the deep learning framework is Pytorch 1.13.1. Among the three types of *Radix Bupleuri* and its adulterants, three samples of each type were randomly taken, totaling nine samples, the bottom end of which was ground flat and placed into Micro-CT for scanning at a scanning set voltage of 45kv, current of 175μA, exposure time of 5s. After scanning and screening, three samples of each type of *Radix Bupleuri*, with 33, 33, and 34 images were selected respectively. A total of 100 images were retained, so there were 300 images for the three types of *Radix Bupleuri*. Those images were exported to DICOM file format, each with a size of approximately 7 M. The DICOM file format is a medical image format that defines the quality and can meet the clinical needs for data exchange, including crucial information such as the original size of the sample, window level, and window width.

### 2.3 Data correction

In the actual Micro-CT scanning process, some defects in the detector itself will have an impact on the scanning results, leading to a particular gap between the ideal situation and pixel response distortion. Therefore, it is necessary to correct the scanned image to restore the actual image to the maximum extent. In this study, geometric correction and light-field correction were performed for the Micro-CT machine used, and the true scanning image was restored to a large extent (Prell et al., 2009).

### 2.4 3D reconstruction

**FIGURE 2:**
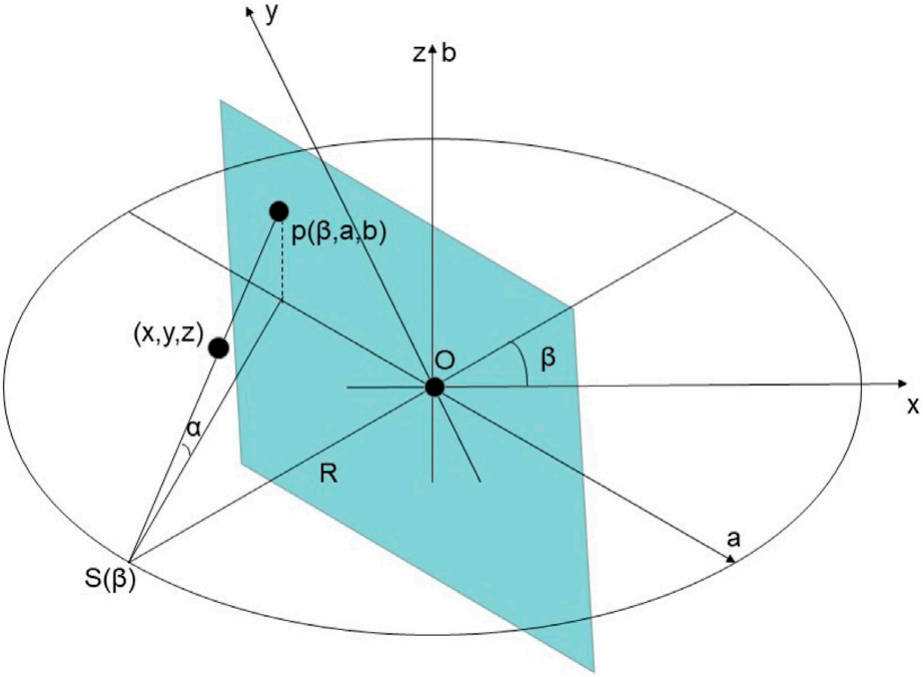
The Schematic of A Conventional Cone-beam CT Scan. Oxyz is the Cartesian coordinate system, and S is the X-ray source, rotating circularly about the *z*-axis with a rotation radius of R, i.e., the distance from the ray source to the center of the sample. The X-rays and their projections can be determined by $p\left(\beta ,a,b\right)$ , where β is an angular parameter.

Micro-CT combined with 3D reconfiguration technology can obtain clear and precise three-dimensional deconstructed structural images of test samples in medicine, biology, geology, etc. (Su and Xiao, 2020). Figure 2 shows the schematic of a conventional cone-beam CT scan. The FDK algorithm is an FBP (2-D fan-beam filtered-back projection) algorithm of the 3-D extension. According to the conical beam CT geometry shown in Figure 2, it can be rewritten as:

$$f\left(x,y,z\right)=\frac{1}{2}\int_{0}^{2\pi }\frac{R^{2}}{{\left(R+xcos\beta +ysin\beta \right)}^{2}}\tilde{p}\left(\beta ,a\left(x,y,\beta \right),b\left(x,y,z,\beta \right)\right)d\beta$$ (1)

$$\tilde{p}\left(\beta ,a,b\right)=\left(\frac{R}{\sqrt{R^{2}+a^{2}+b^{2}}}\cdot p\left(\beta ,a,b\right)\right)*h\left(a\right)$$ (2)

$$a\left(x,y,\beta \right)=R\frac{-xsin\beta +ycos\beta }{R+xcos\beta +ysin\beta }$$ (3)

$$b\left(x,y,z,\beta \right)=\frac{zR}{R+xcos\beta +ysin\beta }$$ (4)

where h(a) is the ramp-filter. The FDK algorithm is implemented in three main steps: data pre-weighting, row-by-row one-dimensional convolution, and weighted three-dimensional back-projection. The FDK algorithm has been widely used because of its simplicity and ease of implementation and has become the most commonly used algorithm in cone-beam reconstruction.

In this study, the FDK algorithm is implemented using ASTRA Toolbox, an open-source toolkit dedicated to reconstructing and analyzing projection data, which is particularly suitable for imaging applications such as computed tomography (CT) (Flouris et al., 2022). The open-source nature of the ASTRA Toolbox allows users to freely access and modify the source code to meet the specific needs of different application scenarios. In addition, the toolkit features powerful GPU acceleration, which significantly improves the computational speed of the FDK algorithm, making it particularly suitable for applications that deal with large-scale projection data and real-time reconstruction (Palenstijn et al., 2017).

In this study, the Micro-CT detector used has a pixel count of 2048 × 2048 with each pixel size of 13.5 µm. In addition, the distance from the X-ray source to the detector is 45.37 cm. These detailed experimental setups provided an accurate basis for the study, ensuring accurate modelling of the Micro-CT system and reliability of the reconstruction process.

### 2.5 Data augmentation

In the process of deep learning, the larger the amount of data, the better the generalization ability of the neural network model, as well as the more stable the ability to classify images. Therefore, the enhancement of images can improve the performance of deep learning models and increase the stability of the model to prevent overfitting. The data enhancement methods adopted in this study included image rotation, image masking, and noise addition.

The image rotation used in this study mainly employs random angle rotation by randomly selecting angles in 90°, 180°, and 270° for clockwise rotation. Taking the center point of the image as the origin $O\left(0,0\right)$ , let the corresponding end of $P\left(x_{0},y_{0}\right)$ after selecting the angle $\theta \;\left(\theta \in \left\{90{}^{\circ},180{}^{\circ},270{}^{\circ}\right\}\right)$ for rotation be $Q\left(x,y\right)$ . The coordinates obtained after clockwise rotation of $P\left(x_{0},y_{0}\right)$ by θ angle are $\left(x_{0}\cos\left(\theta \right)+y_{0}\sin\left(\theta \right),-x_{0}\sin\left(\theta \right)+y_{0}\cos\left(\theta \right)\right)$ and its matrix expression is shown in Equation $\left(5\right)$ . The features of its image do not change before and after rotation.

$$\left[\begin{array}{c} x\\ y\\ 1 \end{array}\right]=\left[-\begin{array}{ccc} \cos\left(\theta \right) & \sin\left(\theta \right) & 0\\ \sin\left(\theta \right) & \cos\left(\theta \right) & 0\\ 0 & 0 & 1 \end{array}\right]\left[\begin{array}{c} x_{0}\\ y_{0}\\ 1 \end{array}\right]$$ (5)

In addition, this study used random masking for data augmentation. An arbitrary point in the image was taken as the starting point; both the length and width were randomly selected between 10pixel and 100pixel, which were used to form a rectangular region, and the gray value of the image within the rectangular area was set to 0, to achieve occlusion of the image.

The last selected data augmentation method is to add noise to the image; in this study, Gaussian noise was added to the DICOM image. Hussain et al. previously added Gaussian noise for data enhancement, and by using Gaussian noise, Gaussian blurring was applied to the images requiring data enhancement, and the accuracy obtained was also improved compared to the previous one (Hussain et al., 2017). The core idea of Gaussian blurring is to replace each pixel value in an image with a weighted average of the pixel values around it, with the weights determined by a Gaussian function. The Gaussian function is the largest at the center and decreases the further away from the center; therefore, pixels closer to the center are weighted more heavily. The Gaussian function has two main parameters: the standard deviation $\left(\sigma \right)$ and pixel value at the center. The core of performing Gaussian blurring is to create a matrix called a filter based on the Gaussian function, the elements of this matrix are the weights calculated by the Gaussian function, and then the filter is applied to each pixel in the image. The pixel values in and around this centroid are weighted and averaged; in this study, the standard deviation $\left(\sigma \right)$ was set to 1.0.

### 2.6 ResNext

A significant advantage of ResNext over ResNet is its ability to improve the model performance without significantly increasing model complexity. By expanding the cardinality number, ResNext can balance the depth of the model and width to a certain extent. A comparison between the network structures of ResNext and ResNet is shown in Table 1.

**TABLE 1:** Network structure comparison table.

Stage	Output	ResNet-50	ResNext-50 $\left(32\times 4d\right)$
Conv1	112×112	7×7 ,64, stride 2	7×7 ,64, stride 2
Conv2	56×56	3×3 max pool, stride 2	3×3 max pool, stride 2
		$\left[\begin{array}{c} 1\times 1,64\\ 3\times 3,64\\ 1\times 1,256 \end{array}\right]\times 3$	$\left[\begin{array}{cc} 1\times 1,128 & \\ 3\times 3,128, & C=32\\ 1\times 1,256 & \end{array}\right]\times 3$
Conv3	28×28	$\left[\begin{array}{c} 1\times 1,128\\ 3\times 3,128\\ 1\times 1,512 \end{array}\right]\times 4$	$\left[\begin{array}{cc} 1\times 1,256 & \\ 3\times 3,256, & C=32\\ 1\times 1,512 & \end{array}\right]\times 4$
Conv4	14×14	$\left[\begin{array}{c} 1\times 1,256\\ 3\times 3,256\\ 1\times 1,1024 \end{array}\right]\times 6$	$\left[\begin{array}{cc} 1\times 1,512 & \\ 3\times 3,512, & C=32\\ 1\times 1,1024 & \end{array}\right]\times 6$
Conv5	7×7	$\left[\begin{array}{c} 1\times 1,512\\ 3\times 3,512\\ 1\times 1,2048 \end{array}\right]\times 3$	$\left[\begin{array}{cc} 1\times 1,1024 & \\ 3\times 3,1024, & C=32\\ 1\times 1,2048 & \end{array}\right]\times 3$
	1×1	global average pool 1000-day fc, softmax	global average pool 1000-day fc, softmax

**FIGURE 3:**
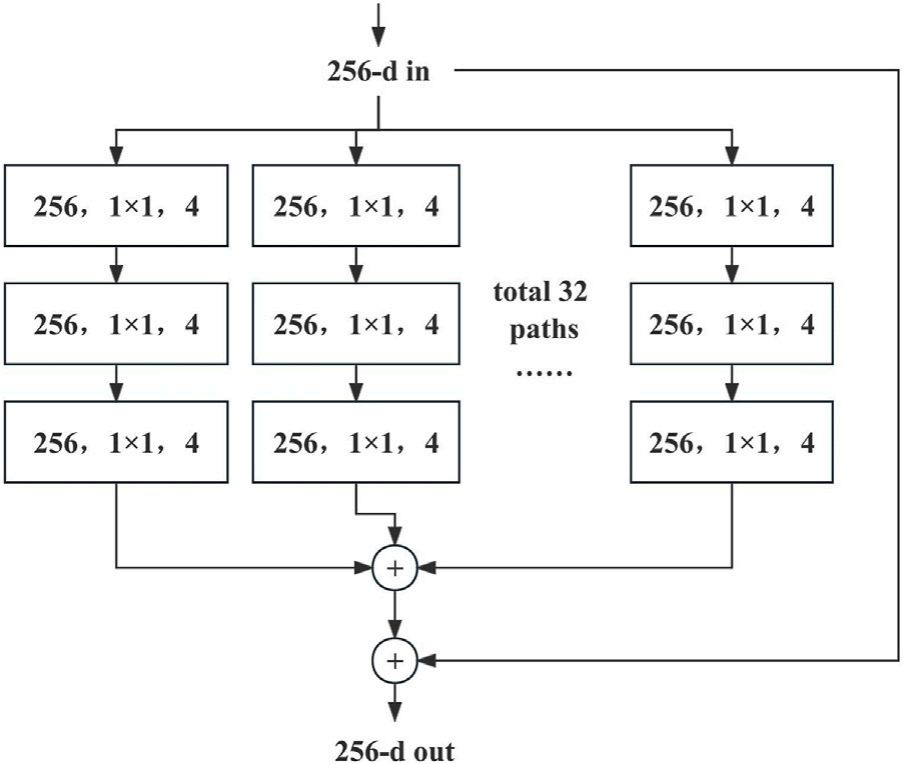
A block of ResNext-50 with cardinality = 32, with roughly the same complexity. The figure illustrates a Bottleneck structure in ResNext-50. The initial input has 256 channels, which undergoes dimensionality reduction to 128 channels using a 1 × 1 convolution. Subsequently, the 128 channels are divided into 32 groups, each containing 4 channels. Finally, dimensionality expansion is performed using a 1 × 1 convolution, resulting in the final output.

As shown in Table 1, the network structure of ResNet-50 is shown on the left, and that of ResNext-50 $\left(32\times 4d\right)$ on the right. The shapes of the residual blocks are in parentheses, the number of stacked blocks is outside the parentheses, and C=32 indicates that the convolution is divided into 32 groups. The Bottleneck structure in ResNext-50 is shown in Figure 3. The Bottleneck module structure enhances the network’s expressive power and computational efficiency by introducing 1×1 convolutions, 3×3 grouped convolutions, and another round of 1×1 convolutions in each module. This design allows the network to maintain lower computational complexity while learning complex features, enabling ResNext-50 to perform exceptionally well in handling large-scale image data and complex tasks. In the original study, ResNext was implemented to improve the accuracy of the model with approximately the same amount of computation as ResNet. Therefore, the deep learning model chosen in this study was ResNext50, and each parameter is listed in Table 2.

**TABLE 2:** Network parameter details.

Parameter	Category/Category/value
Loss Function	Cross-Entropy Loss Function
Optimizer	Stochastic Gradient Descent (SGD)
Activation Function	ReLU
Learning Rate	0.0002
Batch Size	16
epoch	160

In addition, this study used NVIDIA Apex Automatic Mixed Precision (AMP) in the model to improve training efficiency (Micikevicius et al., 2017). AMP allows training and inference to be performed using low-precision floating-point numbers while keeping the model values stable, thus speeding up the training and reducing the memory footprint.

## 3 Results

### 3.1 Results of 3D reconstruction

**FIGURE 4:**
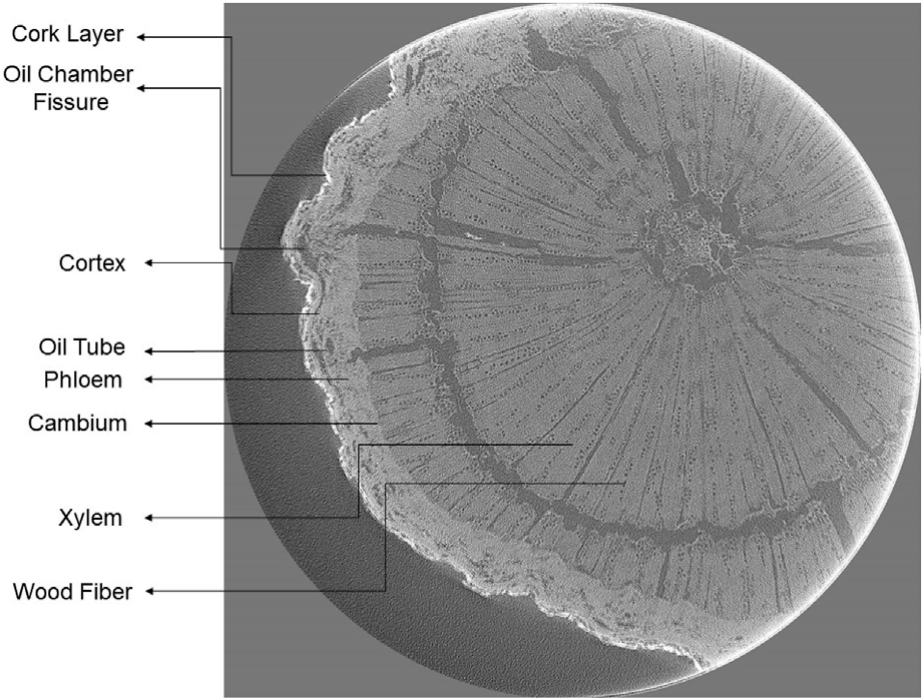
Microstructure of *Radix Bupleuri.* The structures in order from the outside to the inside are: cork layer, cortex, phloem, cambium, and xylem. Among these are oil chamber fissures scattered in the cortex, oil tubes scattered in the phloem, and wood fibers in the xylem.

After 3D reconstruction of the image data in the DICOM file format using the FDK algorithm, a relatively clear tomographic image can be obtained, as shown in Figure 4. Figure 4 shows the tomographic structure of *Radix Bupleuri*, and all structures correspond to the microstructure of *Radix Bupleuri* in the *Identification of Traditional Chinese Medicines* (Feng, 2020). The cork layer is located in the outermost layer, which consists of several rows of cells and plays a protective role; the cortex is located inside the cork layer, and there are oil chamber fissures scattered in the cortex of *Radix Bupleuri*, oil tubes are scattered in the phloem, and the sieve tubes are inconspicuous; the cambium shows a ring-like shape, which consists of three to four rows of parenchyma cells; the ducts of the xylem are sparse and dispersed, and the bundles of wood fibers are arranged in intermittent rings, with radial fissures as xylem rays.

According to the microscopic identification method recorded in *Identification of Traditional Chinese Medicines*, the most apparent difference between North-chaihu and South-chaihu is the number and arrangement of wood fibers in the xylem. The xylem conduits of North-chaihu are sparse and scattered, the wood fiber bundles are arranged in intermittent rings in the middle of the xylem, and the fibers mostly show a polygonal shape. In contrast, the wood fibers in the xylem of South-chaihu are fewer and scattered, and most of them are distributed in the outer part of the xylem.

**FIGURE 5:**
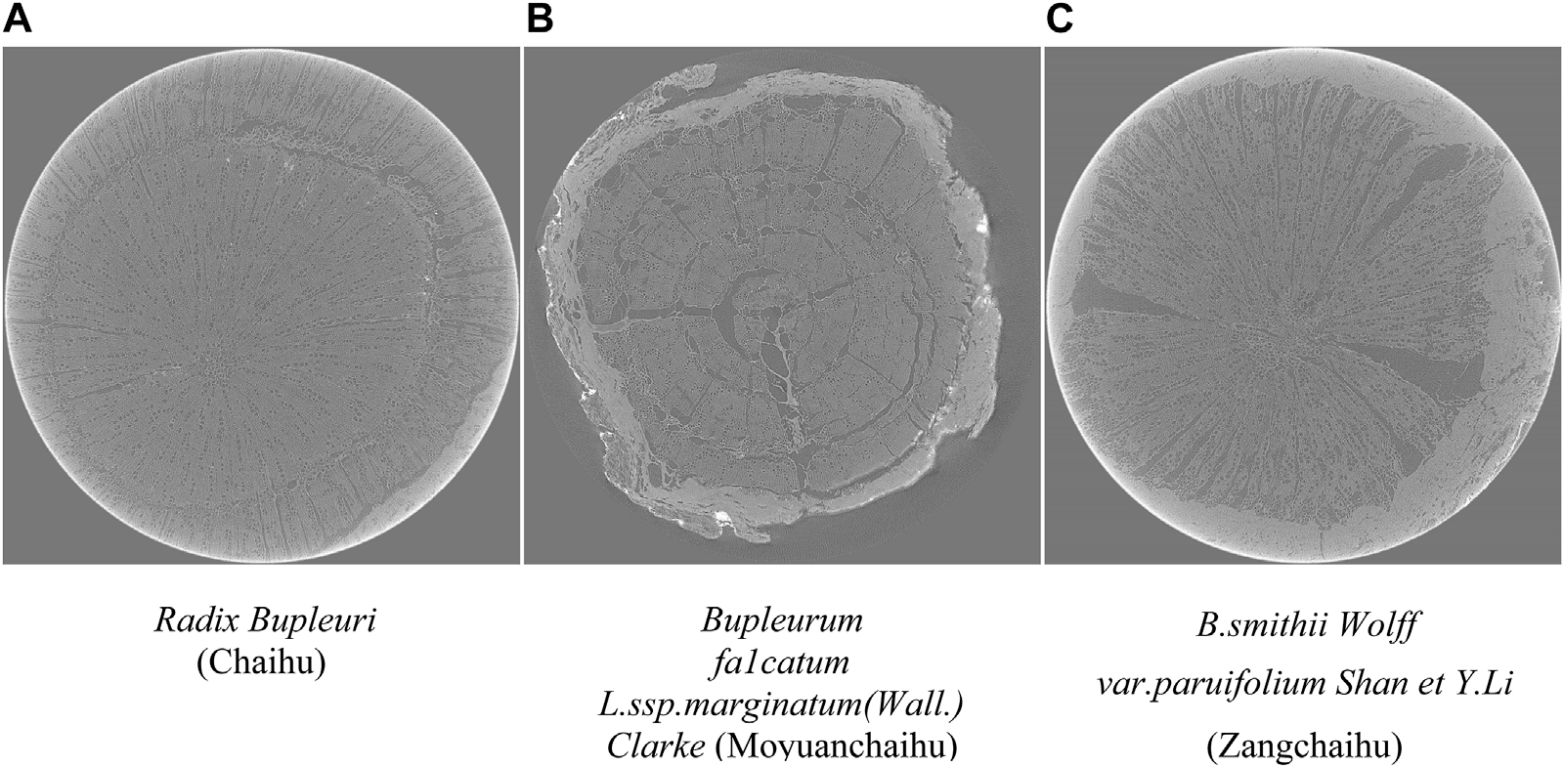
Cross-section of *Radix Bupleuri* and Its Adulterants. The images shown above are all from a single sample and can correspond one-to-one with the optical image in Figure 1. **(A)** shows the microstructure of *Radix Bupleuri* (Chaihu), which is characterized by the interrupted ring-like structure of wood fiber bundles in the xylem. **(B)** shows the microstructure of the *Bupleurum fa1catum L. ssp.marginatum(Wall.) Clarke* (Moyuanchaihu), which is characterized by the aggregation of wood fibers near the formation layer. **(C)** shows the microstructure of *B. smithii Wolff var. paruifolium Shan et Y.Li* (Zangchaihu), characterized by the aggregation of wood fibers in the middle of xylem.

Extending this finding to *Radix Bupleuri* and its adulterants, a significant difference was observed between the wood fibers in the microstructure of *Radix Bupleuri* and its adulterants, as shown in Figure 5. Figure 5A shows the cross-section of the tomography of *Radix Bupleuri*, and it can be clearly seen that the wood fibers in the xylem are arranged in an interrupted ring, which is consistent with the microstructural characteristics of North-chaihu in the book. Figure 5B shows the cross-section of *Bupleurum fa1catum L. ssp.marginatum (Wall.) Clarke* (Moyuanchaihu), whose wood fibers are distributed in a manner that is quite different from that of *Radix Bupleuri*. The wood fibers of Moyuanchaihu have a certain degree of aggregation. The aggregation points are mainly distributed near the formation layer, and the wood fibers in the middle of the xylem are relatively aggregated. Figure 5C shows the cross-section of *B. smithii W. var. paruifolium Shan et Y.Li* (Zangchaihu). Compared with the above two wood fiber distribution patterns, there is a noticeable difference: the wood fibers of Zangchaihu are irregularly arranged in the middle of the xylem, which is more similar to that of the wood fiber of *Radix Bupleuri*, but is more disordered; the most significant difference from the previous two distribution patterns is that the wood fibers of Zangchaihu are mostly aggregated in the middle part of Zangchaihu, which forms a medullary-like part. Based on the above distribution, it can be put into ResNext for training, which can achieve the classification of *Radix Bupleuri* and its adulterants.

### 3.2 Results of the classification

**FIGURE 6:**
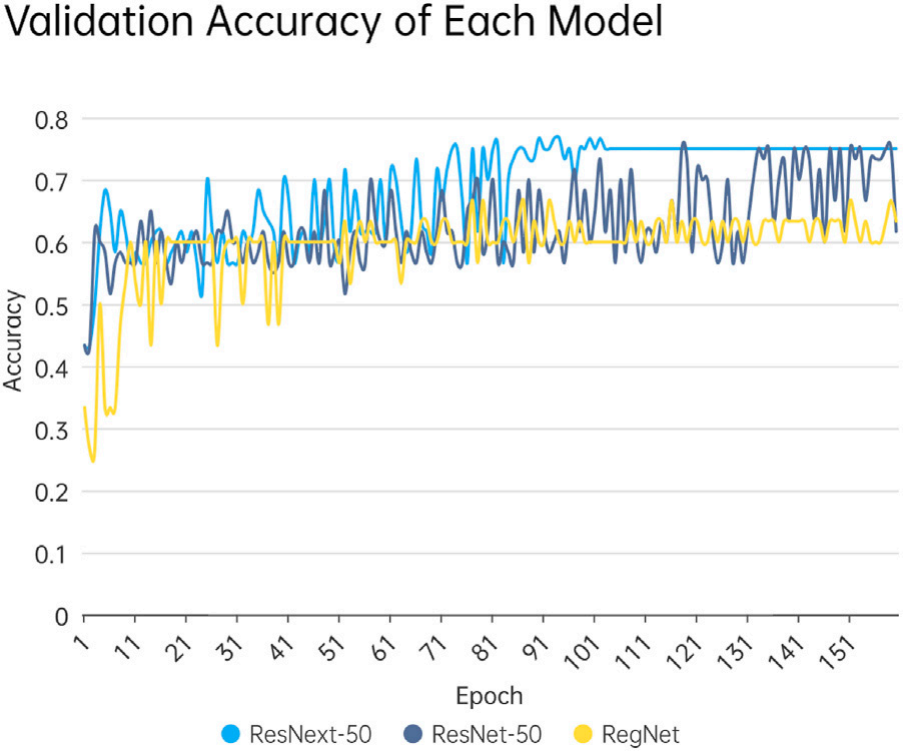
The Iterative Process of Validation Accuracy of Each Model. The horizontal coordinate is the number of epochs and the vertical coordinate is the accuracy. As the number of epochs increases, the accuracy rate increases and stabilizes.

In this study, 1200 DICOM images of 1,024 pixel×1,024 pixel were obtained by data enhancement of the 3D reconstructed DICOM images, of which, 300 images were original images and 900 images were obtained by data enhancement of original images. The training and testing sets were randomly assigned in the ratio of 8:2, i.e., the number of images in the training set was 960, and that of images in the testing set was 240. All the DICOM images were trained and tested using ResNext50, and, a classification accuracy of 75% was obtained. In this study, ResNet-50 and RegNet models were also used to train and validate the same images and compare the results with ResNext-50 model training. The iterative process of validation accuracy of each model is shown in Figure 6.

As shown in Figure 6, the accuracy rate gradually increased with the increase of training epochs. The ResNext-50 model achieves 75% accuracy rate after 70 epochs, stabilizing at this level after 100 epochs. In contrast, ResNet-50, although also capable of reaching 75% accuracy, requires 120 epochs to do so and demonstrates less stability compared to ResNext-50. However, the RegNet network model, which is a classification model with strong generalization ability, does not exhibit satisfactory training results in this study. The validation accuracy increases with the number of epochs and reaches 67% around the 150th epoch.

## 4 Discussion

In this study, data acquisition of *Radix Bupleuri* and its adulterants was carried out by Micro-CT technology, followed by data correction, FDK 3D reconstruction, data augmentation, and finally put into the ResNext50 model for training and testing, which ultimately yielded an accuracy of 75%, and this result can help the doctors to some extent to carry out the research on the identification of *Radix Bupleuri*. In this study, Micro-CT technology was introduced for the identification of *Radix Bupleuri* and its adulterants, which, to a certain extent, made up for the regret that Micro-CT technology was seldom applied in research related to plants and traditional Chinese medicines, and also confirmed the feasibility of using Micro-CT technology in the field of plants and traditional Chinese medicines. After data correction and FDK 3D reconstruction, the DICOM image was clear and obvious so that its microstructures could be distinguished more obviously, and its microscopic features; could be obtained more accurately to identify its authenticity.

Micro-CT has several advantages over electron microscopy: Micro-CT technology does not require slicing of the sample; tomography does not destroy the internal structure of the sample, which allows for the repeated use of the sample and other future studies on the same sample; the sample can be reconstructed in three dimensions if required; Micro-CT technology can be applied to live animals and plants without affecting their growth; Micro-CT can be applied to live animals and plants without affecting their growth; and because the scanning environment of Micro-CT is closed and dark, it can avoid the influence of the surrounding environment on the scanning results.

The significance of this study is to provide a new method for the identification of *Radix Bupleuri* and its adulterants, and the identification and classification of *Radix Bupleuri* and its adulterants were realized based on microscopic features using the ResNext50 model. In general, the images scanned by the Micro-CT technology showed the following microscopic features: the wood fibers in the xylem of *Radix Bupleuri* were arranged in interrupted rings, the wood fibers of Zangchaihu were clustered in the middle, and the wood fibers of Moyuanchaihu were clustered around the forming layer. The results of this study provide a new method for the identification and characterization of *Radix Bupleuri* and even traditional Chinese medicine.

The future work of this study is divided into two main parts. The first part is to use other different deep learning models and optimize these deep learning models to improve the accuracy of model classification. The second part is to scan and classify other kinds of Chinese medicine tablets and their mixed forgeries, and ultimately to realize the ability to classify and identify all Chinese medicine tablets.

## 5 Conclusion

This study highlights the significant potential of Micro-CT technology as a crucial tool in the authentication of Traditional Chinese Medicine (TCM), with a specific focus on *Radix Bupleuri*. The introduction of Micro-CT technology represents a groundbreaking step in providing doctors with an innovative and supplementary diagnostic method for determining the authenticity of medicinal herbs. The capability of Micro-CT to visualize the internal structure of samples offers a unique advantage in discerning microscopic features that aid in the accurate identification of *Radix Bupleuri* and its adulterants.

Moreover, the study recognizes the promising avenue of incorporating deep learning techniques into the analysis of data obtained through Micro-CT scans. Deep learning, with its remarkable achievements in image analysis, is identified as a key direction for future research. The application of deep learning in addressing the authentication challenges of traditional Chinese medicine is anticipated to enhance the precision and efficiency of identification processes. Through the training of neural network models, the potential for automated authentication of TCM is envisioned, providing robust support for the sustainable development of the traditional Chinese medicine industry.

Taken together, the application of Micro-CT technology brings new perspectives and tools for authentication of TCM, whereas the combination of deep learning and other technologies will further enhance the accuracy and efficiency of TCM authentication. This developmental trend is of great significance for the quality control of Chinese medicines. Through more accurate identification methods, the circulation of counterfeit and substandard Chinese medicines can be prevented, and the safety and effectiveness of public medication can be guaranteed. The application of Micro-CT technology also injects new impetus for the sustainable development of the TCM industry. By upgrading the technical level of TCM identification, the TCM industry will become more competitive and provide strong support for the inheritance and innovative development of TCM.

## Data Availability

The datasets presented in this study can be found in online repositories. The names of the repository/repositories and accession number(s) can be found here: https://figshare.com/articles/dataset/_/25239973.
